# Susceptibility Testing of Environmental and Clinical *Aspergillus sydowii* Demonstrates Potent Activity of Various Antifungals

**DOI:** 10.1007/s11046-024-00869-8

**Published:** 2024-07-03

**Authors:** Bram Spruijtenburg, Antonio Rezusta, Jos Houbraken, Ferry Hagen, Theun de Groot, Jacques F. Meis, Eelco F. J. Meijer

**Affiliations:** 1 Radboudumc-CWZ Center of Expertise for Mycology, Weg Door Jonkerbos 100, 6532 SZ Nijmegen, The Netherlands; 2 Canisius-Wilhelmina Hospital (CWZ)/Dicoon, 6532 SZ Nijmegen, The Netherlands; 3 Servicio de Microbiología, Hospital Universitario Miguel Servet, IIS Aragon, Saragossa, Spain; 4 Westerdijk Fungal Biodiversity Institute, Uppsalalaan 8, 3584 CT Utrecht, The Netherlands; 5 Institute for Biodiversity and Ecosystems Dynamics, University of Amsterdam, Amsterdam, The Netherlands; 6 Department of Medical Microbiology, University Medical Center Utrecht, Utrecht, The Netherlands; 7 Institute of Translational Research, Cologne Excellence Cluster On Cellular Stress Responses in Aging-Associated Diseases (CECAD) and Excellence Center for Medical Mycology, University of Cologne, Cologne, Germany

**Keywords:** Aspergillus, Antifungal testing, Antifungal resistance, Aspergillosis, Aspergillus sydowii, Aspergillus versicolor, Aspergillus creber

## Abstract

The genus *Aspergillus* consists of a vast number of medically and environmentally relevant species. *Aspergillus* species classified in series *Versicolores* are ubiquitous in the environment and include the opportunistic pathogen *Aspergillus sydowii*, which is associated with onychomycosis and superficial skin infections. Despite frequent clinical reports of *A. sydowii* and related series *Versicolores* species, antifungal susceptibility data are scarce, hampering optimal treatment choices and subsequent patient outcomes. Here, we employed antifungal susceptibility testing (AFST) based on microbroth dilution on a set of 155 series *Versicolores* strains using the common antifungals amphotericin B, itraconazole, voriconazole, posaconazole, isavuconazole and micafungin with the addition of luliconazole and olorofim. All strains were identified using partial calmodulin gene sequencing, with 145 being *A. sydowii,* seven *A. creber* and three *A. versicolor*, using the latest taxonomic insights. Overall, tested antifungals were potent against the entire strain collection. In comparison to *A. fumigatus*, azole and amphotericin B MICs were slightly elevated for some strains. AFST with luliconazole and olorofim, here reported for the first time, displayed the highest in vitro activity, making these antifungals interesting alternative drugs but clinical studies are warranted for future therapeutic use.

**Supplementary Information:**

The online version contains supplementary material available at 10.1007/s11046-024-00869-8.

## Introduction

*Aspergillus* is one of the most medically important fungal genera, with *A. fumigatus, A. flavus* and *A. terreus* being common etiological agents of infection [1]. The genus is formally divided in subgenera, sections, and series, with the cosmopolitan and environmental species *A. sydowii* classified in series *Versicolores* (subgenus *Nidulantes*, section *Nidulantes*) [2, 3]. *A. sydowii* is an opportunistic non-dermatophytic filamentous fungus with increasing reports of human disease, mainly associated with superficial skin infection and onychomycosis that are often empirically treated with oral terbinafine or itraconazole [4–7]. Cases of keratitis, black grain mycetoma and peritonitis during peritoneal dialysis have also been described [8–11]. Series *Versicolores* species are ubiquitous in the environment [3, 12], and are able to grow in indoor environments, where they pose a health risk, as their spores can aggravate asthma and cause allergies [13]. *A. sydowii* gained attention due to its presence on healthy and diseased corals, and the suggested correlation between increased sea-water temperature due to global warming [14]. Additionally, there are reports off coral aspergillosis and deep-sea colonization [15–17].

Despite its ubiquitous presence, antifungal susceptibility reports of *A. sydowii* and related species are scarce [8, 18, 19]. While only one proven invasive aspergillosis due to *A. sydowii* has been described to date [20], it is more often reported for the closely related *A. versicolor* [21–23]. Morphologically, it is challenging to discriminate *A. sydowii* from related species in the *Versicolores* series [2, 24]. Moreover, the taxonomy of the series underwent significant changes during the last decades, with the most recent change to reduce the series to four species, namely *A. versicolor, A. creber, A. sydowii* and *A. subversicolor* [25]. Nowadays, *Aspergillus* identification relies on sequencing a part of the calmodulin (*CaM*) gene, making accurate identification of *A. sydowii* and related species in the series possible [25, 26]. Here, we perform molecular identification of a large set of 155 environmental and clinical strains, that were preserved as *A. sydowii*, and we performed antifungal susceptibility testing against triazoles, amphotericin B, micafungin, olorofim and luliconazole.

## Material and Methods

### Strains and Media

Fungal strains (n = 155) were obtained from the Westerdijk Fungal Biodiversity Institute (Utrecht, The Netherlands), the microbiology laboratory of Miguel Servet University Hospital (Zaragoza, Spain) and the mycology reference laboratory of the Canisius-Wilhelmina Hospital (Nijmegen, The Netherlands). All strains were previously morphologically and/or molecularly identified as *A. sydowii*. Clinical strains were obtained from patients in the Netherlands and Spain, and environmental strains originated from all continents but Antarctica (Table S1). Strains were preserved in 10% glycerol containing Mueller–Hinton broth at -80 °C. Ethical approval was waived by the local Ethics Committee of Canisius-Wilhelmina Hospital in view of the retrospective nature of the study.

### Molecular Identification

Molecular identification of *A. sydowii* was performed as follows. Strains were inoculated on Sabouraud dextrose agar (SDA, Oxoid, Hampshire, United Kingdom) for seven days at 35 °C. Conidia were suspended into 400 μL Bacterial Lysis Buffer and MagNA Lyser green beads, mechanically lysed for 30 s at 6500 rpm with the MagNA Lyser system (Roche Diagnostics, Mannheim, Germany) and heat-inactivated at 100 °C for 10 min. A 200 μL suspension was used for genomic DNA extraction using the MagNA Pure 96 instrument with the MagNA Pure DNA and Viral NA Small Volume Kit (Roche Diagnostics), following the manufacturer’s instruction as previously described [27]. Amplification and subsequent sequencing of a part of the *CaM* gene using primers Cmd5 5'-CCGAGTACAAGGARGCCTTC-3' and Cmd6 5'-CCGATRGAGGTCATRACGTGG-3' was performed as previously described [28]. In short, amplicons were purified using the AmpliClean protocol (Nimagen, Nijmegen, The Netherlands) followed by a sequencing PCR performed with the BilliantDye mix (Nimagen). Ensuing amplicons were purified according to the D-Pure purification method (Nimagen) and sequenced on a 3500 XL genetic analyzer (Applied Biosystems, Foster City, CA, USA). Calmodulin control sequences of *A. sydowii* (MG991455), *A. versicolor* (OP650543), *A. creber* (LN898757), *A. subversicolor* (ON807889), *A. qilianyuensis* (OM475631), *A. versicolor* (previously known as *A. fructus*) (KX894642), *A. versicolor* (previously known as *A. tabacinus*) (OP244409), *A. creber* (previously known as *A. jensenii*) (OR241157) and *A. versicolor* (previously known as *A. amoenus*) (MK451309) were obtained from the National Center for Biotechnology (NCBI) nucleotide database. *A. nidulans* (MK451456) was included as outgroup. Alignment and phylogenetic tree building was performed with Clustal Omega using the Multiple Alignment Algorithm [29]. Visualization and editing were done with iTOL v6 [30]. Sequences generated in the current study were deposited to NCBI Genbank (accession numbers OR525325–OR525479) (Table S1).

### Antifungal Susceptibility Testing (AFST)

Antifungal susceptibility testing of all strains was performed with broth microdilution using CLSI M38 guidelines [31], and the following drug concentration ranges: amphotericin B 0.016–16 µg/mL (Bristol Meyers Squibb, New York, NY, USA); itraconazole 0.016–16 µg/mL (Janssen Cilag, Breda, the Netherlands); voriconazole 0.016–16 µg/mL (Pfizer, New York, NY, USA); posaconazole 0.016–16 µg/mL (Merck, Darmstadt, Germany); isavuconazole 0.016–16 µg/mL (Basilea Pharmaceutica, Basel, Switzerland); micafungin 0.008–8 µg/mL (Astellas Pharma, Tokyo, Japan); luliconazole 0.001–1 µg/mL (Nihon Nohyaku Co., Tokyo, Japan); olorofim 0.001–1 µg/mL (F2G, Manchester, United Kingdom). Conidia were incubated at a concentration of 0.4 × 10^4^ – 5 × 10^4^ CFU/mL in RPMI1640 medium with antifungal. Minimum inhibitory concentrations (MICs) were visually read after 48 h of incubation at 35 °C as the lowest concentration with a 100% growth reduction when compared to the growth control by two observers, while minimum effective concentrations (MECs) for micafungin were read visually with a microscope as the lowest concentration of drug at which short, stubby, and highly branched hyphae were observed. In addition to broth microdilution, MIC gradient strip testing of amphotericin B (Liofilchem, Roseto degli Abruzzi, Italy) a with concentration gradient of 0.002–32 µg/mL was performed according to manufacturer’s instructions. Spore suspension of 0.5 McFarland was inoculated on the entire surface of the RPMI1640 agar plate (EWC Diagnostics, Steenwijk, The Netherlands) with a sterile cotton swab. MIC gradient strips were placed on the center of the plate and incubated at 35 °C for 48 h. MICs were determined from the inhibition ellipse that intersected with the scale of the strip.

## Results

**Fig. 1:**
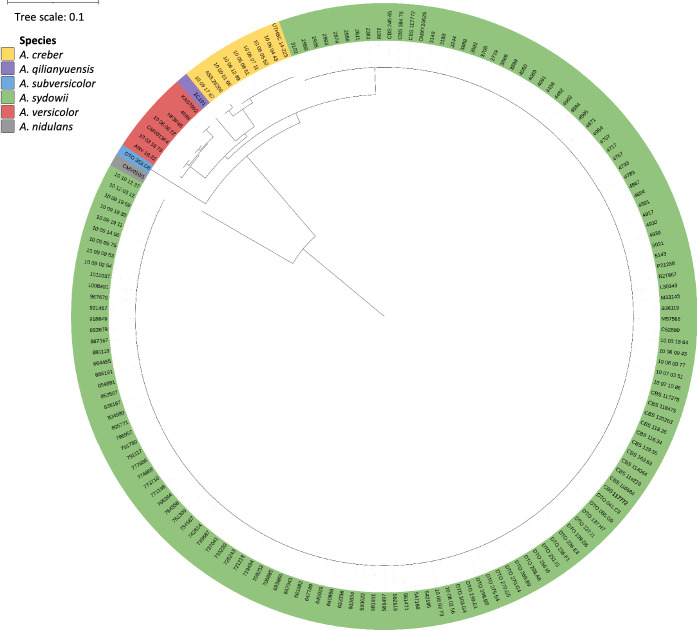
Phylogenetic tree based on multiple sequence alignment of *CaM* sequences. Strains were colored after the closest match to the control species and the tree was rooted to *Aspergillus nidulans*. KAS7992, HP3P46, CMV013F4 and AS3.26206 were identified as *Aspergillus fructus, Aspergillus tabacinus, Aspergillus amoenus* and *Aspergillus jensenii* respectively, using the classification proposed by Jurjević et al. [30]

A total of 155 strains were collected from environmental and clinical sources, mainly from human nails (n = 102) **(**Table 1). Species identification was performed by sequencing part of the calmodulin gene and compared to annotated *A. versicolor*, *A. sydowii*, *A. creber*, *A. qilianyuensis* and *A. subversicolor* strains present in NCBI’s nucleotide database. Multiple sequence alignment of the calmodulin sequences inferred five distinct branches corresponding to *A. sydowii*, *A. versicolor*, *A. creber*, *A. subversicolor* and the outgroup *A. nidulans* respectively (Fig. 1). According to the latest taxonomic insights [25], 145 strains (93.5%) were identified as *A. sydowii,* seven as *A. creber* (4.5%) and three as *A. versicolor* (1.9%) (Table S1). *A. sydowii* was primarily found in nails and indoor dust, *A. creber* from nails and the respiratory tract, and *A. versicolor* was isolated from skin, nails, and an oyster shell. When applying the outdated taxonomy of series *Versicolores* by Jurjević et al. [33], one *A. creber* strain from the Netherlands (10-09-17-67) would be identified as *A. jensenii*, and the three *A. versicolor* strains would be named as *A. fructus* (10–03-18–08; Spain), *A. amoenus* (10–03-18–73; the Netherlands) and *A. tabacinus* (10-06-06-22; the Netherlands).

**Table 1:** Strain summary overview

Country	n (%)
Brazil	1 (0.6)
China	2 (1.3)
Germany	3 (1.9)
Ghana	1 (0.6)
India	1 (0.6)
Indonesia	3 (1.9)
Iran	2 (1.3)
Japan	1 (0.6)
Mexico	2 (1.3)
Micronesia	2 (1.3)
South Africa	1 (0.6)
South Korea	1 (0.6)
Spain	99 (63.9)
The Netherlands	32 (20.6)
Turkey	2 (1.3)
United Kingdom	1 (0.6)
United States	1 (0.6)

Source	n (%)
Clinical	116 (74.8)
Indoor environment	13 (8.4)
Soil or plant associated	6 (3.9)
Miscellaneous*	8 (5.2)
Unknown	12 (7.7)

*Consist of two strains from oyster shells, two from dust, one from cellophane, one from a djembe, one from tattoo ink and one specified as non-clinical

In vitro AFST according to CLSI M38 guidelines was performed on all strains using amphotericin B, four triazoles, micafungin, olorofim and luliconazole. MICs were comparable between the series *Versicolores* species and differed 2 two-fold dilutions at most (Table S1)*.* Luliconazole and olorofim demonstrated the highest in vitro activities based on the lowest MIC_90_ of both 0.008 µg/mL, with MICs ranging from ≤ 0.001 to 0.0063 µg/mL (Table 2). Out of the four tested triazole agents, itraconazole and posaconazole showed higher in vitro activity than voriconazole and isavuconazole with the former two displaying a MIC_90_ of 0.5 µg/mL and the latter a MIC_90_ of 2 µg/mL. Overall, with the exception for amphotericin B in some cases, all tested agents showed potent activity. For strains with amphotericin B MICs at the lower or upper range (0.125, 0.25 and 2 µg/mL), an MIC gradient strip was performed in addition to 20 randomly selected strains (Table S1). MICs of all tested strains were within 2 two-fold dilutions, with the highest MIC for two strains of 3 µg/mL, which corresponds to an MIC of 2 µg/mL, according to CLSI guidelines.

**Table 2:** In vitro AFST of *Aspergillus sydowii* (n = 145), according to M38 guidelines

Antifungal	Range (µg/mL)	Mode (µg/mL)	GM (µg/mL)	MIC_50_ (µg/mL)	MIC_90_ (µg/mL)
Amphotericin B^&^	0.125–2	1	0.909	1	2
Itraconazole	0.063–1	0.5	0.361	0.5	0.5
Voriconazole	0.063–4	1	0.878	1	2
Posaconazole	0.031–1	0.25	0.203	0.25	0.5
Isavuconazole	0.125–4	0.5	0.714	1	2
Micafungin*	≤ 0.008	≤ 0.008	0.009	≤ 0.008	≤ 0.008
Luliconazole	≤ 0.001–0.031	0.004	0.004	0.004	0.008
Olorofim	≤ 0.001–0.063	0.004	0.004	0.004	0.008

^&^Results verified by MIC gradient strip (n = 47, Range 0.25–3)

*Minimum effective concentrations (MECs) were used for micafungin. GM, geometric mean; MIC, minimal inhibitory concentration

## Discussion

Non-dermatophyte mould onychomycosis caused by *Aspergillus* species is emerging, especially in diabetic populations and the elderly [31]. *A. sydowii* has been described as a causative agent in onychomycosis for decades, while the closely related species *A. versicolor* is also reported as cause of invasive disease [22, 23, 32]. Series *Versicolores* species are phenotypically similar, but *A. sydowii* can be distinguished from the other species by its production of blue-green to turquoise conidia, in combination with growth at 35 °C. Small reduced diminutive conidial heads, which resemble penicillate conidiophores, are commonly produced in *A. sydowii* strains, but can also be present in other series *Versicolores* species [25]. Based on morphology, *A. sydowii* is often misidentified as *A. versicolor,* or due to the production of green coloured colonies and penicillate conidiophores, as *Penicillium* spp. colonization or contamination [5]. For reliable identification, especially for clinical strains that can have deviating phenotype, a sequence-based approach is recommended. Molecular identification using ITS sequencing does not contain sufficient variation to discriminate closely related species [25]. Hence calmodulin sequencing is recommended as a secondary identification marker in identifying *A. sydowii*. Although *A. sydowii* may grow at 37 °C, invasive pulmonary aspergillosis or disseminated disease has rarely been described to date. One study reported *A. sydowii* from blood and lung biopsies suggesting invasive disease [19], and another study found a single case of invasive pulmonary aspergillosis [20].

In the current study, a large collection of 155 *Aspergillus* series *Versicolores* strains were identified based on calmodulin sequencing. While the series *Versicolores* underwent numerous taxonomic changes, the latest taxonomic insights are applied here, which divides the series in four species supported by extensive phylogenetic data, namely *A. sydowii, A. versicolor, A. creber* and *A. subversicolor* [25]. Previous taxonomic studies divided the series into 17 species, including many cryptic ones, complicating identification in clinical practices [33]. Here, *A. sydowii* is found to be highly prevalent in clinical samples, mainly involved in onychomycosis, as reported before [6]. *A. creber* and *A. versicolor* were isolated from similar sources such as nails, skin or the respiratory tract, all of which are exposed to aerosols frequently contain spores of *Versicolores* species [24].

As to date, antifungal susceptibility testing results of *A. sydowii* are rarely reported, with 20 strains tested at most in one study where the authors report elevated MICs of amphotericin B, with a MIC_90_ of 8 µg/mL [19]. In our study, MICs of amphotericin B were several dilutions lower, despite both AFST were performed according to CLSI M38 guidelines [31]. With CLSI microbroth dilution, we found a MIC_90_ of 2 µg/mL for amphotericin B (range 0.125–2). Because of this discrepancy, we decided to add a second method to verify our results. Using MIC gradient strips, MICs were comparable and were all within 2 two-fold dilution. The highest MIC found with MIC gradient strips was 3 µg/mL, where CLSI microbroth dilution was 2 µg/mL. Minor variability is often observed when MICs from different laboratories are compared. Although no MIC gradient strips for amphotericin B were previously performed, comparisons between CLSI and MIC gradient strip in *Aspergillus* species yielded overall high agreements in MICs [34, 35], as was also found in the current study.

For triazoles, itraconazole and posaconazole displayed a higher in vitro activity than voriconazole and isavuconazole, which is comparable with previous studies on *A. sydowii* and other *Aspergillus* species [36, 37]. When compared to *A. fumigatus* epidemiological cutoff values (ECVs), voriconazole and isavuconazole MICs seem elevated [38]. Interestingly, species from the *Versicolores* series are ubiquitous in the environment, whereas azole fungicides are extensively used in agricultural settings [39]. Azole use in agriculture has driven the emergence of resistant *Aspergillus* strains, especially in *A. fumigatus*, but high azole MICs could not be found in this cohort [40].

To the best of our knowledge, antifungal activity of luliconazole and olorofim against species from the *Versicolores* series are reported for the first time. Both drugs displayed excellent potency, which might be interesting alternatives depending on clinical disease. Luliconazole is an imidazole available in the USA to treat onchomycosis and dermatophytic fungi [41]. Onychomycosis treatment generally consists of systemic therapy with terbinafine or itraconazole, while luliconazole can be applied topically. Given that *A. sydowii* and related species from the *Versicolores* series are the causative agent of onychomycosis, luliconazole might be an interesting option, but clinical studies are warranted. Olorofim is the first orotomide antifungal drug, currently evaluated in a phase III clinical trial [42, 43]. The drug has good activity against numerous *Aspergillus* species, including azole resistant *A. fumigatus* isolates [44, 45]. As MICs of olorofim were low for *A. sydowii,* the drug can be considered if azole resistance would develop or when standard therapeutic options are unavailable for lung or systemic infection.

To summarize, we identified a large collection of 145 *A. sydowii,* seven *A. creber* and three *A. versicolor* strains based on calmodulin sequencing according to latest taxonomic insights. AFST was subsequently performed on all isolated according to CLSI guidelines against amphotericin B, several azoles, micafungin and olorofim. All antifungals displayed potent activity against all strains, with luliconazole and olorofim exhibiting the highest in vitro activity.

### Supplementary Information

Below is the link to the electronic supplementary material.

Supplementary file1 (DOCX 35 kb)
