# Supplementary material for: Susceptibility Testing of Environmental and Clinical Aspergillus sydowii Demonstrates Potent Activity of Various Antifungals
Source: Mycopathologia. 2024 Jul 3;189(4):61. doi: 10.1007/s11046-024-00869-8 (PMC11222195; doi:10.1007/s11046-024-00869-8)
Supplement: Supplementary file 1 — Supplementary file1 (DOCX 35 kb) [file 11046_2024_869_MOESM1_ESM.docx]

**Supplementary**

**Table S1: Overview of 155 strains including country of origin and MICs according to microbroth dilution CLSI M38 3^rd^ edition guidelines.** MICs in µg/mL.

| **ID** | **Country** | **Source** | **Species** | **AMB (microbroth)** | **AMB (MIC gradient strip)** | **ITC** | **VOR** | **POS** | **ISA** | **MFG** | **LULI** | **OLF** | ***CaM* Genbank accession no.** |
| --- | --- | --- | --- | --- | --- | --- | --- | --- | --- | --- | --- | --- | --- |
| CBS 117772 | South Korea | Soil from pepper field | *A. sydowii* | 1 | 0.75 | 0.5 | 2 | 1 | 2 | ≤0.008 | 0.008 | 0.004 | OR525325 |
| CBS 384.75 | India | Usar soil | *A. sydowii* | 1 | N.D. | 0.5 | 1 | 0.25 | 1 | ≤0.008 | 0.004 | 0.002 | OR525326 |
| CBS 245.65 | United States | Cellophane | *A. sydowii* | 1 | 1.5 | 0.25 | 0.5 | 0.125 | 0.5 | ≤0.008 | 0.004 | 0.004 | OR525327 |
| 2198 | Spain | Nail | *A. sydowii* | 1 | 1 | 0.5 | 2 | 0.25 | 1 | ≤0.008 | 0.004 | 0.008 | OR525328 |
| 2381 | Spain | Nail | *A. sydowii* | 1 | N.D. | 0.5 | 1 | 0.25 | 0.5 | ≤0.008 | 0.004 | 0.008 | OR525329 |
| 2611 | Spain | Nail | *A. sydowii* | 1 | 1 | 0.5 | 1 | 0.25 | 1 | ≤0.008 | 0.004 | 0.008 | OR525330 |
| 2658 | Spain | Nail | *A. sydowii* | 2 | 1.5 | 0.25 | 0.5 | 0.125 | 0.5 | ≤0.008 | 0.002 | 0.008 | OR525331 |
| 2674 | Spain | Nail | *A. sydowii* | 1 | N.D. | 0.5 | 0.5 | 0.125 | 0.5 | ≤0.008 | 0.002 | 0.008 | OR525332 |
| 2863 | Spain | Nail | *A. sydowii* | 1 | N.D. | 0.5 | 2 | 0.25 | 2 | ≤0.008 | 0.002 | 0.008 | OR525333 |
| 2935 | Spain | Nail | *A. sydowii* | 1 | N.D. | 0.25 | 1 | 0.25 | 1 | ≤0.008 | 0.004 | 0.008 | OR525334 |
| 2959 | Spain | Nail | *A. sydowii* | 1 | N.D. | 0.5 | 1 | 0.25 | 1 | ≤0.008 | 0.002 | 0.008 | OR525335 |
| 3102 | Spain | Nail | *A. sydowii* | 1 | N.D. | 0.5 | 4 | 1 | 4 | ≤0.008 | 0.008 | 0.008 | OR525336 |
| 3149 | Spain | Nail | *A. sydowii* | 1 | N.D. | 0.5 | 1 | 0.25 | 1 | ≤0.008 | 0.004 | 0.004 | OR525337 |
| 3188 | Spain | Nail | *A. sydowii* | 0.5 | N.D. | 0.5 | 2 | 0.25 | 1 | ≤0.008 | 0.004 | 0.004 | OR525338 |
| 3244 | Spain | Nail | *A. sydowii* | 1 | N.D. | 0.5 | 1 | 0.25 | 1 | ≤0.008 | 0.004 | 0.004 | OR525339 |
| 3689 | Spain | Nail | *A. sydowii* | 1 | N.D. | 0.5 | 1 | 0.25 | 1 | ≤0.008 | 0.004 | 0.008 | OR525340 |
| 3692 | Spain | Nail | *A. sydowii* | 1 | N.D. | 1 | 1 | 0.125 | 1 | ≤0.008 | 0.002 | 0.004 | OR525341 |
| 3709 | Spain | Nail | *A. sydowii* | 1 | N.D. | 0.5 | 1 | 0.25 | 1 | ≤0.008 | 0.004 | 0.008 | OR525342 |
| 3719 | Spain | Nail | *A. sydowii* | 2 | 1.5 | 0.5 | 2 | 0.5 | 2 | ≤0.008 | 0.008 | 0.008 | OR525343 |
| 3869 | Spain | Nail | *A. sydowii* | 1 | N.D. | 1 | 0.5 | 0.25 | 0.5 | ≤0.008 | 0.002 | 0.004 | OR525344 |
| 3888 | Spain | Nail | *A. sydowii* | 0.25 | 0.5 | 0.25 | 0.25 | 0.063 | 0.5 | ≤0.008 | ≤0.001 | 0.016 | OR525345 |
| 4050 | Spain | Nail | *A. sydowii* | 1 | N.D. | 0.5 | 1 | 0.25 | 0.5 | ≤0.008 | 0.004 | 0.008 | OR525346 |
| 4089 | Spain | Nail | *A. sydowii* | 0.5 | N.D. | 0.25 | 1 | 0.063 | 0.25 | ≤0.008 | ≤0.001 | 0.002 | OR525347 |
| 4091 | Spain | Nail | *A. sydowii* | 1 | N.D. | 0.5 | 0.5 | 0.125 | 1 | ≤0.008 | 0.002 | 0.002 | OR525348 |
| 4456 | Spain | Nail | *A. sydowii* | 2 | 2 | 0.5 | 1 | 0.25 | 1 | ≤0.008 | 0.004 | 0.004 | OR525349 |
| 4482 | Spain | Nail | *A. sydowii* | 2 | 2 | 0.5 | 0.5 | 0.125 | 2 | ≤0.008 | 0.002 | 0.004 | OR525350 |
| 4560 | Spain | Nail | *A. sydowii* | 2 | 1.5 | 0.25 | 1 | 0.25 | 2 | ≤0.008 | 0.002 | 0.004 | OR525351 |
| 4584 | Spain | Nail | *A. sydowii* | 1 | N.D. | 0.25 | 2 | 0.125 | 0.5 | ≤0.008 | 0.004 | 0.008 | OR525352 |
| 4585 | Spain | Nail | *A. sydowii* | 1 | N.D. | 0.5 | 2 | 0.5 | 1 | ≤0.008 | 0.004 | 0.008 | OR525353 |
| 4671 | Spain | Nail | *A. sydowii* | 1 | N.D. | 0.25 | 0.5 | 0.125 | 0.5 | ≤0.008 | 0.002 | 0.004 | OR525354 |
| 4698 | Spain | Nail | *A. versicolor^1^* | 1 | N.D. | 0.125 | 0.5 | 0.125 | 0.5 | 0.063 | 0.002 | 0.016 | OR525355 |
| 4964 | Spain | Nail | *A. sydowii* | 1 | N.D. | 0.5 | 1 | 0.25 | 1 | ≤0.008 | 0.002 | 0.004 | OR525356 |
| 4707 | Spain | Nail | *A. sydowii* | 1 | N.D. | 0.5 | 0.5 | 0.125 | 1 | ≤0.008 | 0.002 | 0.004 | OR525357 |
| 4717 | Spain | Nail | *A. sydowii* | 1 | N.D. | 0.5 | 1 | 0.25 | 0.5 | ≤0.008 | 0.004 | 0.002 | OR525358 |
| 4767 | Spain | Nail | *A. sydowii* | 1 | N.D. | 0.5 | 1 | 0.125 | 0.5 | ≤0.008 | 0.002 | 0.004 | OR525359 |
| 4793 | Spain | Nail | *A. sydowii* | 0.5 | N.D. | 0.5 | 0.5 | 0.125 | 0.5 | ≤0.008 | ≤0.001 | 0.004 | OR525360 |
| 4789 | Spain | Nail | *A. sydowii* | 0.5 | N.D. | 0.25 | 1 | 0.25 | 0.5 | ≤0.008 | 0.004 | 0.004 | OR525361 |
| 4867 | Spain | Nail | *A. sydowii* | 1 | N.D. | 0.25 | 1 | 0.125 | 0.5 | ≤0.008 | 0.002 | 0.004 | OR525362 |
| 4884 | Spain | Nail | *A. sydowii* | 1 | N.D. | 0.5 | 1 | 0.25 | 1 | ≤0.008 | 0.004 | 0.004 | OR525363 |
| 4891 | Spain | Nail | *A. sydowii* | 1 | 1 | 0.5 | 2 | 0.5 | 2 | ≤0.008 | 0.004 | 0.008 | OR525364 |
| 4917 | Spain | Nail | *A. sydowii* | 1 | N.D. | 0.5 | 2 | 0.5 | 1 | ≤0.008 | 0.004 | 0.004 | OR525365 |
| 4930 | Spain | Nail | *A. sydowii* | 1 | N.D. | 0.5 | 1 | 0.25 | 0.5 | ≤0.008 | 0.002 | 0.004 | OR525366 |
| 4938 | Spain | Nail | *A. sydowii* | 2 | 1 | 0.25 | 0.5 | 0.25 | 0.5 | ≤0.008 | ≤0.001 | 0.004 | OR525367 |
| 5021 | Spain | Nail | *A. sydowii* | 1 | N.D. | 0.25 | 1 | 0.25 | 1 | ≤0.008 | 0.002 | 0.002 | OR525368 |
| 5143 | Spain | Nail | *A. sydowii* | 1 | N.D. | 0.25 | 1 | 0.25 | 0.5 | ≤0.008 | 0.002 | 0.004 | OR525369 |
| P21288 | Spain | Nail | *A. sydowii* | 1 | N.D. | 0.5 | 2 | 0.5 | 1 | ≤0.008 | 0.004 | 0.008 | OR525370 |
| B27967 | Spain | Nail | *A. sydowii* | 2 | N.D. | 0.5 | 1 | 0.25 | 1 | ≤0.008 | 0.004 | 0.008 | OR525371 |
| L30349 | Spain | Nail | *A. sydowii* | 1 | N.D. | 1 | 1 | 0.25 | 1 | ≤0.008 | 0.004 | 0.004 | OR525372 |
| M33143 | Spain | Nail | *A. sydowii* | 1 | N.D. | 0.5 | 2 | 0.5 | 2 | ≤0.008 | 0.008 | 0.004 | OR525373 |
| B36119 | Spain | Nail | *A. sydowii* | 1 | N.D. | 0.5 | 2 | 0.5 | 2 | ≤0.008 | 0.008 | 0.008 | OR525374 |
| M57566 | Spain | Nail | *A. sydowii* | 1 | N.D. | 0.5 | 2 | 0.25 | 1 | ≤0.008 | 0.004 | 0.004 | OR525375 |
| C62880 | Spain | Nail | *A. sydowii* | 1 | N.D. | 0.25 | 0.5 | 0.125 | 0.5 | ≤0.008 | 0.002 | 0.002 | OR525376 |
| 10-03-18-64 | The Netherlands | Oyster shell | *A. sydowii* | 1 | 0.75 | 0.25 | 0.125 | 0.125 | 0.125 | ≤0.008 | ≤0.001 | 0.002 | OR525377 |
| 10-03-18-73 | The Netherlands | Oyster shell | *A. versicolor^2^* | 2 | 2 | 0.25 | 0.5 | 0.125 | 0.5 | 0.031 | 0.002 | 0.002 | OR525378 |
| 10-06-04-43 | The Netherlands | Nail | *A. creber* | 2 | N.D. | 0.125 | 0.25 | 0.125 | 0.5 | 0.031 | ≤0.001 | 0.002 | OR525379 |
| 10-06-05-52 | The Netherlands | Maxillary sinus | *A. creber* | 1 | 1 | 0.5 | 1 | 0.25 | 0.25 | 0.125 | 0.002 | 0.016 | OR525380 |
| 10-06-06-22 | The Netherlands | Skin | *A. versicolor^3^* | 0.5 | 2 | 0.5 | 1 | 0.5 | 1 | 0.063 | 0.004 | 0.004 | OR525381 |
| 10-06-07-11 | The Netherlands | Nail | *A. creber* | 0.5 | N.D. | 0.125 | 0.5 | 0.063 | 0.25 | 0.063 | ≤0.001 | ≤0.001 | OR525382 |
| 10-06-08-61 | The Netherlands | BAL | *A. creber* | 1 | N.D. | 0.125 | 0.5 | 0.125 | 0.5 | 0.031 | ≤0.001 | 0.002 | OR525383 |
| 10-06-09-43 | The Netherlands | Sputum | *A. sydowii* | 1 | N.D. | 0.125 | 0.25 | 0.125 | 0.25 | ≤0.008 | ≤0.001 | ≤0.001 | OR525384 |
| 10-06-09-77 | The Netherlands | BAL | *A. sydowii* | 0.5 | 0.25 | 0.063 | 0.063 | 0.031 | 0.125 | ≤0.008 | ≤0.001 | 0.002 | OR525385 |
| 10-06-12-89 | The Netherlands | Nail | *A. creber* | 1 | N.D. | 1 | 0.5 | 0.25 | 0.25 | ≤0.008 | 0.004 | ≤0.001 | OR525386 |
| 10-07-03-51 | The Netherlands | External ear canal | *A. sydowii* | 0.125 | 0.25 | 0.063 | 0.25 | 0.031 | 0.25 | ≤0.008 | 0.002 | 0.002 | OR525387 |
| 10-07-10-86 | The Netherlands | Sputum | *A. sydowii* | 1 | 1.5 | 0.5 | 1 | 0.5 | 1 | ≤0.008 | 0.004 | 0.004 | OR525388 |
| CBS 117278 | Ghana | Djembe | *A. sydowii* | 1 | N.D. | 0.5 | 1 | 0.25 | 1 | ≤0.008 | 0.004 | 0.008 | OR525389 |
| CBS 118475 | The Netherlands | Tattoo ink | *A. sydowii* | 1 | N.D. | 0.5 | 1 | 0.25 | 1 | ≤0.008 | 0.004 | 0.008 | OR525390 |
| CBS 120263 | China | Man | *A. sydowii* | 1 | N.D. | 0.25 | 0.5 | 0.125 | 0.5 | ≤0.008 | 0.002 | 0.004 | OR525391 |
| CBS 118.26 | China | Red rice | *A. sydowii* | 0.25 | 0.5 | 0.25 | 0.5 | 0.125 | 1 | ≤0.008 | ≤0.001 | 0.002 | OR525392 |
| CBS 116.34 | Japan | Tobacco | *A. sydowii* | 0.5 | N.D. | 0.5 | 1 | 0.25 | 0.5 | ≤0.008 | 0.002 | ≤0.001 | OR525393 |
| CBS 129.55 | Brazil | Sputum | *A. sydowii* | 1 | N.D. | 0.25 | 1 | 0.125 | 0.5 | ≤0.008 | 0.002 | 0.002 | OR525394 |
| CBS 169.63 | The Netherlands | Unknown | *A. sydowii* | 1 | N.D. | 0.5 | 0.5 | 0.125 | 0.25 | ≤0.008 | 0.004 | 0.004 | OR525395 |
| CBS 114064 | Germany | Wallpaper surface | *A. sydowii* | 2 | 0.5 | 0.5 | 4 | 0.5 | 2 | ≤0.008 | 0.008 | 0.016 | OR525396 |
| CBS 114219 | United Kingdom | Environment | *A. sydowii* | 0.5 | N.D. | 0.5 | 1 | 0.5 | 1 | ≤0.008 | 0.004 | 0.004 | OR525397 |
| CBS 116684 | The Netherlands | Milled rice | *A. sydowii* | 1 | N.D. | 0.5 | 2 | 0.25 | 2 | ≤0.008 | 0.004 | 0.008 | OR525398 |
| CBS 170.63 | South Africa | Silage | *A. sydowii* | 0.25 | 0.25 | 0.5 | 0.5 | 0.063 | 1 | ≤0.008 | ≤0.001 | 0.004 | OR525399 |
| DTO 041-C3 | The Netherlands | Indoor air | *A. sydowii* | 1 | N.D. | 1 | 2 | 0.5 | 2 | ≤0.008 | 0.008 | 0.008 | OR525400 |
| DTO 055-G9 | The Netherlands | Indoor air | *A. sydowii* | 2 | 3 | 0.5 | 2 | 0.5 | 2 | ≤0.008 | 0.008 | 0.004 | OR525401 |
| DTO 137-H7 | The Netherlands | Insulation material | *A. sydowii* | 1 | N.D. | 0.5 | 1 | 0.25 | 1 | ≤0.008 | 0.002 | 0.002 | OR525402 |
| DTO 137-I1 | Germany | Indoor environment | *A. sydowii* | 1 | N.D. | 0.25 | 0.5 | 0.125 | 0.5 | ≤0.008 | 0.002 | 0.002 | OR525403 |
| DTO 139-D6 | Germany | Indoor environment | *A. sydowii* | 2 | 2 | 0.5 | 2 | 0.5 | 1 | ≤0.008 | 0.008 | 0.008 | OR525404 |
| DTO 236-E4 | Indonesia | Dust | *A. sydowii* | 0.5 | N.D. | 0.25 | 0.5 | 0.125 | 0.5 | ≤0.008 | 0.002 | 0.004 | OR525405 |
| DTO 236-F1 | Indonesia | Dust | *A. sydowii* | 1 | N.D. | 0.5 | 1 | 0.25 | 1 | ≤0.008 | 0.004 | 0.004 | OR525406 |
| DTO 251-I3 | Iran | Clinical specimen | *A. sydowii* | 2 | 1 | 0.5 | 2 | 0.25 | 1 | ≤0.008 | 0.002 | 0.004 | OR525407 |
| DTO 266-I6 | Micronesia | House dust | *A. sydowii* | 1 | N.D. | 0.25 | 0.5 | 0.125 | 0.5 | ≤0.008 | 0.002 | 0.002 | OR525408 |
| DTO 269-A8 | Micronesia | House dust | *A. sydowii* | 1 | N.D. | 0.25 | 0.25 | 0.125 | 0.25 | ≤0.008 | ≤0.001 | 0.002 | OR525409 |
| DTO 269-B9 | Indonesia | House dust | *A. sydowii* | 1 | N.D. | 0.5 | 1 | 0.25 | 1 | ≤0.008 | 0.004 | 0.004 | OR525410 |
| DTO 270-G4 | Mexico | House dust | *A. sydowii* | 1 | N.D. | 0.5 | 2 | 0.5 | 2 | ≤0.008 | 0.016 | 0.008 | OR525411 |
| DTO 270-G5 | Mexico | House dust | *A. sydowii* | 1 | N.D. | 0.5 | 2 | 0.5 | 2 | ≤0.008 | 0.016 | 0.008 | OR525412 |
| DTO 275-E4 | Iran | Nail | *A. sydowii* | 1 | N.D. | 0.25 | 0.25 | 0.125 | 0.25 | ≤0.008 | 0.002 | ≤0.001 | OR525413 |
| DTO 299-B8 | Turkey | Indoor hospital air | *A. sydowii* | 1 | N.D. | 0.25 | 0.5 | 0.125 | 0.5 | ≤0.008 | 0.002 | 0.004 | OR525414 |
| DTO 299-E1 | Turkey | Indoor hospital air | *A. sydowii* | 2 | 3 | 0.5 | 2 | 0.5 | 2 | ≤0.008 | 0.016 | ≤0.001 | OR525415 |
| DTO 303-G4 | The Netherlands | Unknown | *A. sydowii* | 1 | N.D. | 0.25 | 0.5 | 0.125 | 0.5 | ≤0.008 | 0.002 | 0.004 | OR525416 |
| 10-08-02-56 | The Netherlands | Sputum | *A. sydowii* | 1 | 0.75 | 0.5 | 2 | 0.25 | 1 | ≤0.008 | 0.004 | 0.004 | OR525417 |
| 10-08-02-73 | The Netherlands | Sputum | *A. sydowii* | 1 | 1 | 0.5 | 2 | 0.5 | 1 | ≤0.008 | 0.004 | 0.004 | OR525418 |
| 542195 | Spain | Nail | *A. sydowii* | 2 | 3 | 0.25 | 0.5 | 0.125 | 0.5 | ≤0.008 | 0.002 | 0.002 | OR525419 |
| 547168 | Spain | Nail | *A. sydowii* | 2 | 1 | 0.5 | 1 | 0.25 | 0.5 | ≤0.008 | 0.002 | 0.004 | OR525420 |
| 561471 | Spain | Nail | *A. sydowii* | 1 | N.D. | 0.5 | 0.5 | 0.125 | 0.5 | ≤0.008 | 0.002 | 0.004 | OR525421 |
| 562118 | Spain | Nail | *A. sydowii* | 0.5 | N.D. | 0.5 | 0.5 | 0.125 | 0.5 | ≤0.008 | 0.002 | 0.004 | OR525422 |
| 581497 | Spain | Nail | *A. sydowii* | 1 | N.D. | 0.5 | 2 | 0.25 | 1 | ≤0.008 | 0.008 | 0.004 | OR525423 |
| 581931 | Spain | Nail | *A. sydowii* | 1 | 1 | 0.25 | 1 | 0.125 | 0.5 | ≤0.008 | 0.002 | 0.002 | OR525424 |
| 593622 | Spain | Nail | *A. sydowii* | 0.5 | N.D. | 0.25 | 2 | 0.125 | 0.5 | ≤0.008 | 0.004 | 0.002 | OR525425 |
| 602634 | Spain | Nail | *A. sydowii* | 1 | N.D. | 0.5 | 2 | 0.25 | 2 | ≤0.008 | 0.008 | 0.008 | OR525426 |
| 633336 | Spain | Nail | *A. sydowii* | 1 | N.D. | 0.25 | 1 | 0.25 | 0.5 | ≤0.008 | 0.008 | 0.002 | OR525427 |
| 640986 | Spain | Nail | *A. sydowii* | 0.5 | N.D. | 0.5 | 1 | 0.25 | 0.5 | ≤0.008 | 0.002 | 0.002 | OR525428 |
| 645026 | Spain | Nail | *A. sydowii* | 2 | 2 | 0.5 | 2 | 0.25 | 2 | ≤0.008 | 0.008 | 0.008 | OR525429 |
| 647789 | Spain | Nail | *A. sydowii* | 1 | N.D. | 0.125 | 0.25 | 0.063 | 0.125 | ≤0.008 | ≤0.001 | 0.004 | OR525430 |
| 661882 | Spain | Nail | *A. sydowii* | 1 | N.D. | 0.5 | 1 | 0.25 | 1 | ≤0.008 | 0.004 | 0.004 | OR525431 |
| 667945 | Spain | Nail | *A. sydowii* | 1 | N.D. | 0.5 | 1 | 0.25 | 1 | ≤0.008 | 0.004 | 0.004 | OR525432 |
| 683866 | Spain | Nail | *A. sydowii* | 1 | N.D. | 0.5 | 1 | 0.25 | 0.5 | ≤0.008 | 0.002 | 0.002 | OR525433 |
| 706885 | Spain | Nail | *A. sydowii* | 1 | N.D. | 0.25 | 1 | 0.063 | 0.5 | ≤0.008 | 0.004 | 0.002 | OR525434 |
| 709233 | Spain | Nail | *A. sydowii* | 0.5 | N.D. | 0.25 | 1 | 0.25 | 0.5 | ≤0.008 | 0.002 | 0.004 | OR525435 |
| 719494 | Spain | Nail | *A. sydowii* | 0.5 | 1 | 0.25 | 0.5 | 0.125 | 0.25 | ≤0.008 | 0.002 | 0.002 | OR525436 |
| 721218 | Spain | Nail | *A. sydowii* | 0.5 | N.D. | 0.5 | 0.5 | 0.125 | 0.25 | ≤0.008 | 0.002 | 0.002 | OR525437 |
| 725243 | Spain | Nail | *A. sydowii* | 0.5 | N.D. | 0.25 | 0.5 | 0.25 | 0.5 | ≤0.008 | 0.004 | 0.002 | OR525438 |
| 733220 | Spain | Nail | *A. sydowii* | 1 | N.D. | 0.125 | 0.25 | 0.125 | 0.25 | ≤0.008 | 0.002 | 0.004 | OR525439 |
| 737041 | Spain | Nail | *A. sydowii* | 0.5 | N.D. | 0.25 | 0.5 | 0.125 | 0.5 | ≤0.008 | 0.002 | 0.002 | OR525440 |
| 739687 | Spain | Nail | *A. sydowii* | 2 | 1 | 0.5 | 2 | 0.25 | 1 | ≤0.008 | 0.004 | 0.016 | OR525441 |
| 742814 | Spain | Nail | *A. sydowii* | 1 | N.D. | 0.25 | 1 | 0.125 | 1 | ≤0.008 | 0.002 | 0.002 | OR525442 |
| 754587 | Spain | Nail | *A. sydowii* | 0.5 | N.D. | 0.5 | 1 | 0.25 | 1 | ≤0.008 | 0.004 | 0.002 | OR525443 |
| 761309 | Spain | Nail | *A. sydowii* | 1 | N.D. | 0.5 | 1 | 0.25 | 1 | ≤0.008 | 0.004 | 0.004 | OR525444 |
| 764358 | Spain | Nail | *A. sydowii* | 1 | N.D. | 0.25 | 0.5 | 0.25 | 0.5 | ≤0.008 | 0.002 | 0.002 | OR525445 |
| 766264 | Spain | Nail | *A. sydowii* | 1 | N.D. | 0.5 | 2 | 0.25 | 0.5 | ≤0.008 | 0.004 | 0.004 | OR525446 |
| 771196 | Spain | Nail | *A. sydowii* | 1 | 1 | 0.5 | 2 | 0.25 | 1 | ≤0.008 | 0.004 | 0.008 | OR525447 |
| 773710 | Spain | Nail | *A. sydowii* | 1 | N.D. | 0.5 | 2 | 0.25 | 1 | ≤0.008 | 0.004 | 0.004 | OR525448 |
| 774869 | Spain | Nail | *A. sydowii* | 1 | N.D. | 0.5 | 1 | 0.25 | 1 | ≤0.008 | 0.004 | 0.002 | OR525449 |
| 777886 | Spain | Nail | *A. sydowii* | 1 | N.D. | 0.125 | 0.5 | 0.125 | 0.25 | ≤0.008 | 0.002 | 0.004 | OR525450 |
| 791117 | Spain | Nail | *A. sydowii* | 0.5 | N.D. | 0.5 | 1 | 0.125 | 1 | ≤0.008 | 0.002 | 0.002 | OR525451 |
| 791780 | Spain | Nail | *A. sydowii* | 0.5 | N.D. | 0.5 | 1 | 0.25 | 0.5 | ≤0.008 | ≤0.001 | 0.002 | OR525452 |
| 796957 | Spain | Nail | *A. sydowii* | 1 | N.D. | 0.25 | 1 | 0.125 | 1 | ≤0.008 | 0.002 | 0.002 | OR525453 |
| 805771 | Spain | Nail | *A. sydowii* | 1 | N.D. | 0.5 | 1 | 0.5 | 1 | ≤0.008 | 0.004 | 0.002 | OR525454 |
| 834599 | Spain | Nail | *A. sydowii* | 1 | N.D. | 0.5 | 1 | 0.25 | 1 | ≤0.008 | 0.004 | 0.004 | OR525455 |
| 835167 | Spain | Nail | *A. sydowii* | 1 | 1.5 | 0.5 | 0.5 | 0.125 | 0.5 | ≤0.008 | 0.002 | 0.004 | OR525456 |
| 853507 | Spain | Nail | *A. sydowii* | 0.5 | N.D. | 0.125 | 1 | 0.125 | 0.5 | ≤0.008 | 0.002 | 0.008 | OR525457 |
| 854881 | Spain | Nail | *A. sydowii* | 0.5 | N.D. | 0.25 | 0.5 | 0.25 | 0.5 | ≤0.008 | 0.004 | 0.002 | OR525458 |
| 856191 | Spain | Nail | *A. sydowii* | 2 | 2 | 0.5 | 0.5 | 0.125 | 0.5 | ≤0.008 | 0.002 | 0.004 | OR525459 |
| 864455 | Spain | Nail | *A. sydowii* | 0.5 | N.D. | 0.5 | 1 | 0.125 | 0.5 | ≤0.008 | 0.004 | 0.004 | OR525460 |
| 881119 | Spain | Sputum | *A. sydowii* | 0.5 | N.D. | 0.25 | 0.5 | 0.125 | 0.5 | ≤0.008 | 0.002 | 0.004 | OR525461 |
| 887167 | Spain | Nail | *A. sydowii* | 0.5 | 0.75 | 0.5 | 0.25 | 1 | 0.5 | ≤0.008 | 0.031 | 0.063 | OR525462 |
| 893679 | Spain | Nail | *A. sydowii* | 0.5 | N.D. | 0.125 | 0.5 | 0.125 | 0.5 | ≤0.008 | 0.002 | 0.004 | OR525463 |
| 918649 | Spain | Nail | *A. sydowii* | 0.5 | N.D. | 0.25 | 1 | 0.125 | 1 | ≤0.008 | 0.004 | 0.002 | OR525464 |
| 921457 | Spain | Nail | *A. sydowii* | 2 | 1.5 | 0.25 | 1 | 0.25 | 0.5 | ≤0.008 | 0.004 | 0.004 | OR525465 |
| 967679 | Spain | Nail | *A. sydowii* | 1 | N.D. | 0.125 | 0.5 | 0.125 | 0.5 | ≤0.008 | 0.004 | ≤0.001 | OR525466 |
| 1008491 | Spain | Nail | *A. sydowii* | 1 | N.D. | 0.5 | 1 | 0.25 | 1 | ≤0.008 | 0.004 | 0.004 | OR525467 |
| 1011097 | Spain | Nail | *A. sydowii* | 1 | N.D. | 0.5 | 1 | 0.25 | 1 | ≤0.008 | 0.004 | 0.004 | OR525468 |
| 10-09-01-66 | The Netherlands | Unknown | *A. creber* | 0.5 | 0.25 | 0.5 | 0.5 | 0.125 | 0.25 | 0.125 | ≤0.001 | ≤0.001 | OR525469 |
| 10-09-02-64 | The Netherlands | Unknown | *A. sydowii* | 0.25 | 0.25 | 0.125 | 0.25 | 0.125 | 0.25 | ≤0.008 | 0.008 | 0.008 | OR525470 |
| 10-09-09-63 | The Netherlands | Unknown | *A. sydowii* | 2 | 0.5 | 0.5 | 1 | 0.25 | 0.5 | ≤0.008 | 0.004 | 0.004 | OR525471 |
| 10-09-09-75 | The Netherlands | Unknown | *A. sydowii* | 0.5 | N.D. | 0.5 | 2 | 0.5 | 2 | ≤0.008 | 0.016 | ≤0.001 | OR525472 |
| 10-09-14-06 | The Netherlands | Unknown | *A. sydowii* | 2 | 2 | 0.125 | 0.5 | 0.125 | 0.5 | ≤0.008 | 0.002 | 0.016 | OR525473 |
| 10-09-17-67 | The Netherlands | Unknown | *A. creber^4^* | 1 | N.D. | 0.5 | 2 | 0.5 | 1 | 0.063 | 0.008 | 0.008 | OR525474 |
| 10-09-19-11 | The Netherlands | Unknown | *A. sydowii* | 1 | 1 | 0.5 | 0.5 | 0.25 | 1 | ≤0.008 | 0.004 | 0.002 | OR525475 |
| 10-09-19-30 | The Netherlands | Unknown | *A. sydowii* | 0.5 | N.D. | 0.125 | 0.5 | 0.125 | 0.25 | ≤0.008 | 0.002 | 0.008 | OR525476 |
| 10-09-19-69 | The Netherlands | Unknown | *A. sydowii* | 1 | 1.5 | 0.125 | 1 | 0.125 | 0.25 | ≤0.008 | 0.002 | 0.004 | OR525477 |
| 10-10-12-37 | The Netherlands | Unknown | *A. sydowii* | 0.5 | 0.75 | 0.125 | 0.25 | 0.063 | 0.5 | ≤0.008 | ≤0.001 | 0.008 | OR525478 |
| 10-12-03-12 | The Netherlands | External ear canal | *A. sydowii* | 1 | 0.75 | 0.25 | 1 | 0.25 | 0.5 | ≤0.008 | 0.004 | 0.002 | OR525479 |

^1^formerly known as *A. fructus*, ^2^formerly known as *A. amoenus*, ^3^formerly known as *A. tabacinus*, ^4^formerly known as *A. jensenii*. AMB, amphotericin B; ITC, itraconazole; VOR, voriconazole; POS, posaconazole; ISA, isavuconazole; MFG, micafungin; LULI, luliconazole; OLF, olorofim; *CaM*, calmodulin; BAL, Bronchoalveolar lavage; N.D.; not determined.
